# Automated procedure assessing the accuracy of HRCT–PET registration applied in functional virtual bronchoscopy

**DOI:** 10.1186/s13550-021-00810-w

**Published:** 2021-07-26

**Authors:** Gábor Opposits, Marianna Nagy, Zoltán Barta, Csaba Aranyi, Dániel Szabó, Attila Makai, Imre Varga, László Galuska, Lajos Trón, László Balkay, Miklós Emri

**Affiliations:** 1 Division of Nuclear Medicine and Translational Imaging, Department of Medical Imaging, Faculty of Medicine, University of Debrecen, Nagyerdei krt. 98., Debrecen, 4032 Hungary; 2 Division of Radiology and Imaging Science, Department of Medical Imaging, Faculty of Medicine, University of Debrecen, Nagyerdei krt. 98., Debrecen, 4032 Hungary; 3 Department of Pulmonology, Faculty of Medicine, University of Debrecen, Nagyerdei krt. 98., Debrecen, 4032 Hungary

**Keywords:** Computed tomography, Diagnostics, Image registration, Image segmentation, Image-guided bronchoscopy

## Abstract

**Background:**

Bronchoscopy serves as direct visualisation of the airway. Virtual bronchoscopy provides similar visual information using a non-invasive imaging procedure(s). Early and accurate image-guided diagnosis requires the possible highest performance, which might be approximated by combining anatomical and functional imaging. This communication describes an advanced functional virtual bronchoscopic (fVB) method based on the registration of PET images to high-resolution diagnostic CT images instead of low-dose CT images of lower resolution obtained from PET/CT scans. PET/CT and diagnostic CT data were collected from 22 oncological patients to develop a computer-aided high-precision fVB. Registration of segmented images was performed using elastix.

**Results:**

For virtual bronchoscopy, we used an in-house developed segmentation method. The quality of low- and high-dose CT image registrations was characterised by expert’s scoring the spatial distance of manually paired corresponding points and by eight voxel intensity-based (dis)similarity parameters. The distribution of (dis)similarity parameter correlating best with anatomic scoring was bootstrapped, and 95% confidence intervals were calculated separately for acceptable and insufficient registrations. We showed that mutual information (MI) of the eight investigated (dis)similarity parameters displayed the closest correlation with the anatomy-based distance metrics used to characterise the quality of image registrations. The 95% confidence intervals of the bootstrapped MI distribution were [0.15, 0.22] and [0.28, 0.37] for ﻿insufficient and acceptable registrations, respectively. In case of any new patient, a calculated MI value of registered low- and high-dose CT image pair within the [0.28, 0.37] or the [0.15, 0.22] interval would suggest acceptance or rejection, respectively, serving as an aid for the radiologist.

**Conclusion:**

A computer-aided solution was proposed in order to reduce reliance on radiologist’s contribution for the approval of acceptable image registrations.

## Background

There is a growing interest in increasing the diagnostic yield in investigating peripheral pulmonary lesions and malignant tracheal tumours [1–4]. Visualisation of the structure of the thorax and lung and combining it with taking tissue samples is of primary importance. For this purpose, bronchoscopy is commonly used; it provides an optical image of the inner surface of the bronchi using fibre optics and thus guides sampling.

The disadvantage of bronchoscopic examination is that it provides visual information on details within the bronchus only, but not the outside extension of tumour tissue (suggesting how to orient the sampling needle), and it is invasive. Virtual bronchoscopy (VB) is a non-invasive procedure and offers visualisation of the airway from the in- and outside along with its neighbouring blood vessels and lymph nodes, derived from computed tomography (CT) and/or positron emission tomography (PET) [3, 5–7]. Additionally, there are further imaging modalities capable of providing three-dimensional images of the bronchial tree and lung, including, among others, ultrasound [8, 9].

The PET method excels from others due to its ability to give information about the tissue biochemistry. The fluorodeoxyglucose (FDG) accumulation map displays the intensity distribution of carbohydrate metabolism; thus, reports on tumour suspect localisation(s). FDG-PET studies have been proven to be useful in the selection of benign from malignant lesions [10–13] and staging [14–17].

Acquisition of simultaneous information about the structure and function implies a presumable improvement in the diagnostic yield. The 3D rendering of 18 F-FDG PET/CT images was first used for virtual bronchoscopy in 2006 [18]. Unfortunately, the majority, if not all of the few publications reporting on the use of PET imaging in VB, applied low-resolution ldCT images obtained from PET/CT scan. Thus, this kind of VB using both CT and PET lacked the high resolution offered by the diagnostic CT images. The high resolution in this combined type of VB can be assured by substituting ldCT with hdCT, but it involves complex registration problems.

Registration of PET images to ldCT images is remarkably more straightforward than that to hdCT because of the identical geometry [19]. We decided to work out an image processing pipeline based on high-resolution hdCT images to perform precise registration of PET images to diagnostic CT images for functional virtual bronchoscopy (fVB). Thus, a radiologist expert was asked to validate and decide on the accept/reject issue in the initial phase of the project. To spare the contribution of the radiologist expert, we developed a computer-aided method to evaluate the hdCT image registration to the ldCT image allowing the performance of high-resolution CT-PET-based fVB.

## Methods

### Patients

PET/CT and hdCT scans were performed on 22 oncological patients (11 females and 11 males) aged between 42 and 81 years (mean 63 years, median 63 years, SD 9 years). The Regional and Institutional Ethics Committee at our university approved this clinical study, which was then carried out under the relevant guidelines and regulations. Clinical data of the patients were collected from medical records and entered into a patient database.

### Imaging protocols

The hdCT images were constructed by a Siemens Emotion 16 camera (Siemens Healthcare GmbH, Erlangen, Germany). These investigations were carried out with 110 kVp voltage and pitch 0.8. Other hospitals also sent hdCT data obtained by Philips Brilliance 10 (Koninklijke Philips N.V., Amsterdam, Netherlands) with 120 kVp and pitch 0.9, or Siemens Emotion Duo (Siemens Healthcare GmbH, Erlangen, Germany) with 110 kVp and pitch 0.8. The ldCT and PET scans were performed by a PET/CT camera Philips GEMINI TF TOF 64 (Koninklijke Philips N.V., Amsterdam, Netherlands) applying 120 kVp and pitch 0.829. Scans were performed using automatic exposure control (AEC).

The ldCT and hdCT imaging was carried out with less than 1-year time difference. Scan conditions were not standardised; hands of the subjects were along the trunk during the ldCT scan, while in case of hdCT they were over the head, making ldCT–hdCT registration more difficult. Also, there was no air retention constraint during the ldCT scan (PET/CT) as opposed to hdCT. Scan time and the imaged region of the body were not uniform either.

### Registration and segmentation

#### Registration

Performing VB, functional information on the living tissues can be anatomically localised by projecting the adequate radioactivity accumulation to the surface of hdCT segmented organs. To transfer functional information to the space of high-performance hdCT (reference frame), the hdCT and ldCT images must be anatomically fitted by image registration as PET, and ldCT images are inherently fitted due to the identical geometry of the appropriate scans.

For image registration, we used the *elastix* algorithm [20, 21] based on Insight Toolkit [22], which supports both rigid and non-rigid body transformations. Having completed the registration of the ldCT and hdCT images of the patients, a radiologist expert categorised the registration of the image pairs as good or bad in the initial phase of the project.

#### Segmentation

**Fig. 1:**
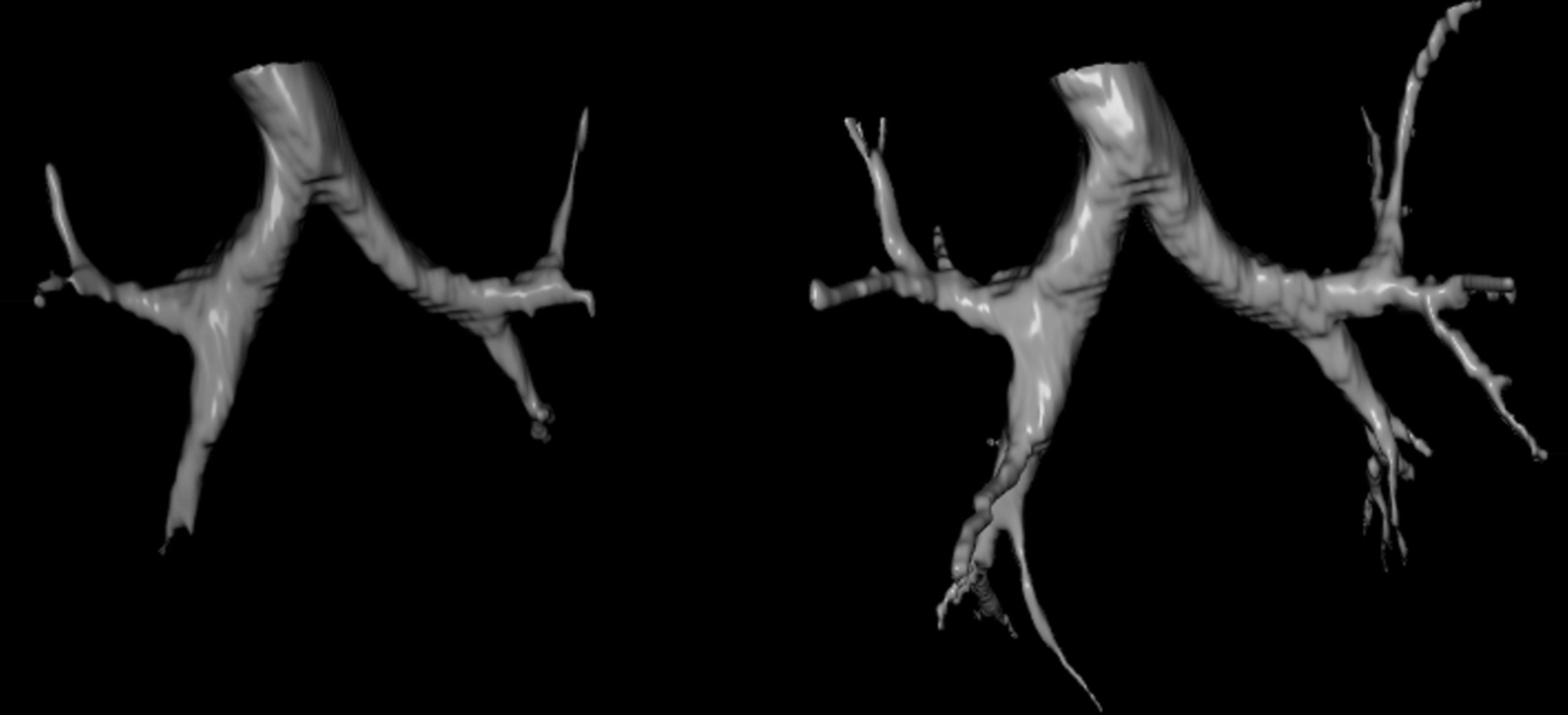
Surface model of airway images segmented from ldCT (left) and hdCT (right) images

The aim was to establish a dedicated fVB protocol with the simultaneous visualisation of both anatomical and functional information. To have a more structured image of the airway, one should work with the hdCT image. Figure 1 shows the remarkable difference between the surface model of the segmented airway images obtained from low-dose and high-dose CT scans of the chest.

The high-dose CT image of the airway offers more detailed structural data for VB with higher-generation branches. Thus, high-quality fVB necessitates the registration of PET image to hdCT. As previously explained, this registration can be performed using the transformation matrix of the ldCT to hdCT registration. To obtain precise registration of ldCT and hdCT images, only the essential part of the data should be used excluding irrelevant details, e.g. bed and ribs. The relevant data can be selected by constructing and using an appropriate lung mask. A segmented hdCT lung image was obtained with the help of the M-SEGM (see “Appendix”) algorithm, used after selecting voxels with intensities between − 1000 and − 400 HU representing bronchial airway and parenchyma. As a first step, the intensity value of the non-segmented voxels of the lung image was set to zero, while that of the segmented ones was set to one, adding up to a lung-mask (referred to as the “lung-mask”). This procedure was followed by constructing lung-masked ldCT and hdCT images with voxel intensities set to the product of the “lung-mask” intensity values and that of the ldCT or hdCT. The registration of the ldCT and hdCT images was performed using the lung-masked images.

Sometimes, the rib cage may be well aligned to the detriment of vessel structures near the border of the lung [20, 21], occasionally causing the relocation of the lung apex to the abdomen region in the hdCT image. Successful registration of lung-masked ldCT and hdCT images using elastix was not possible because the software inevitably stopped. We implemented a specific application to get a functioning lung-mask, eliminating all major discontinuities and adding the mediastinal voxels to the lung-mask. The attachment of the mediastinal region was necessary because a part of the lung-masked ldCT image frequently overlapped the hdCT mediastinum, which was eliminated from the lung-masked hdCT image. The obtained mask is referred to as “functioning lung-mask.” Using this application elastix worked satisfactorily with the default input parameters [20, 21].

The bronchial tree segmentation from the ldCT and hdCT was performed using modified GeoS 2.2 Microsoft software [23], or alternately our region-growing segmentation (multi-SEGMentation: M-SEGM a part of M3I framework [24]) method, a voxel intensity-based iterative procedure (to be published elsewhere). In addition to the application in the current project, M-SEGM can be used to solve similar tasks. Our segmentation procedure also has the advantage that the necessary modification can be easily solved for the actual local needs. M-SEGM allows automatic segmentation having specified a parameter (seed point) and the homogeneity criterion for the tissue to be segmented (airway, blood vessel or parenchyma, etc.). The application of M-SEGM enabled the segmentation of the hdCT tracheal tree up to the origin of the sub-segment bronchi.

#### Construction of airway tree and surface model

**Fig. 2:**
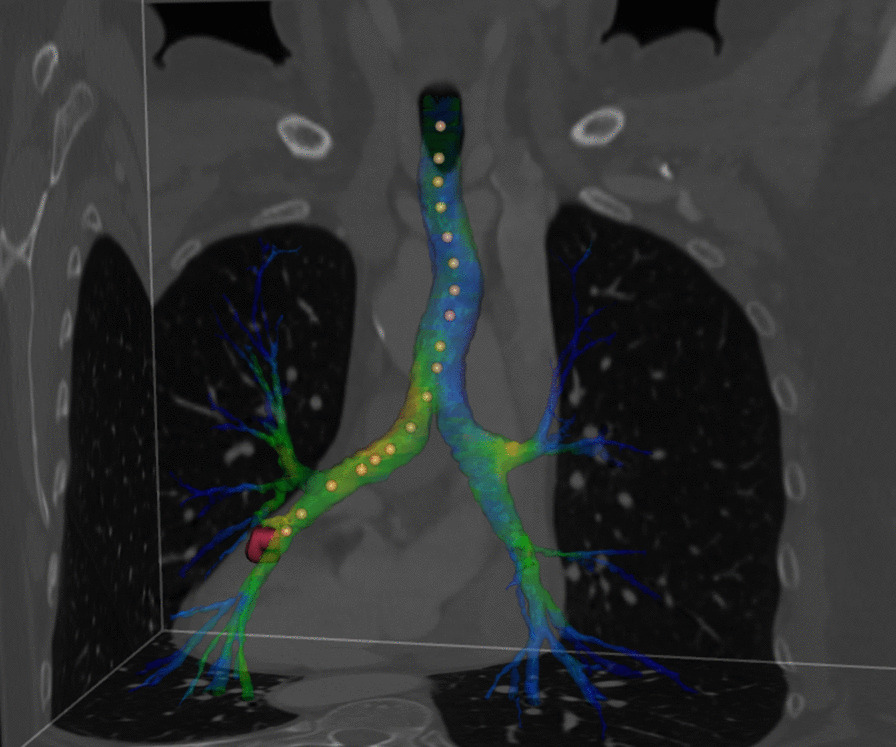
Parts of the three orthogonal sections of the thorax and the segmented bronchial tree comprising a possible traversal (indicated by dots) in the  3D surface model

The ensemble of the segmented airway voxels can be used to generate their centre of gravity slice by slice (centre points are located in the air compartment). The total of the centre of gravity points will be referred to as airway tree or skeleton (dotted line in Fig. 2). The individual points of the skeleton are characterised by a set of indices reporting on the ordinal number (*i*) and the coordinates of the points (*x*, *y*, *z*) and the ordinal number of the first adjacent point(s) along the new branch(es) (*i*1, *i*2, *i*3…) also indicating the number of branches at the particular point (the format of an element in the skeleton file: (*i*, *x*, *y*, *z*, *i*1, *i*2, *i*3…). After its creation, the skeleton was cleansed by erasing short branches containing less than six voxels unless they comprise a new branch before the sixth point.

**Fig. 3:**
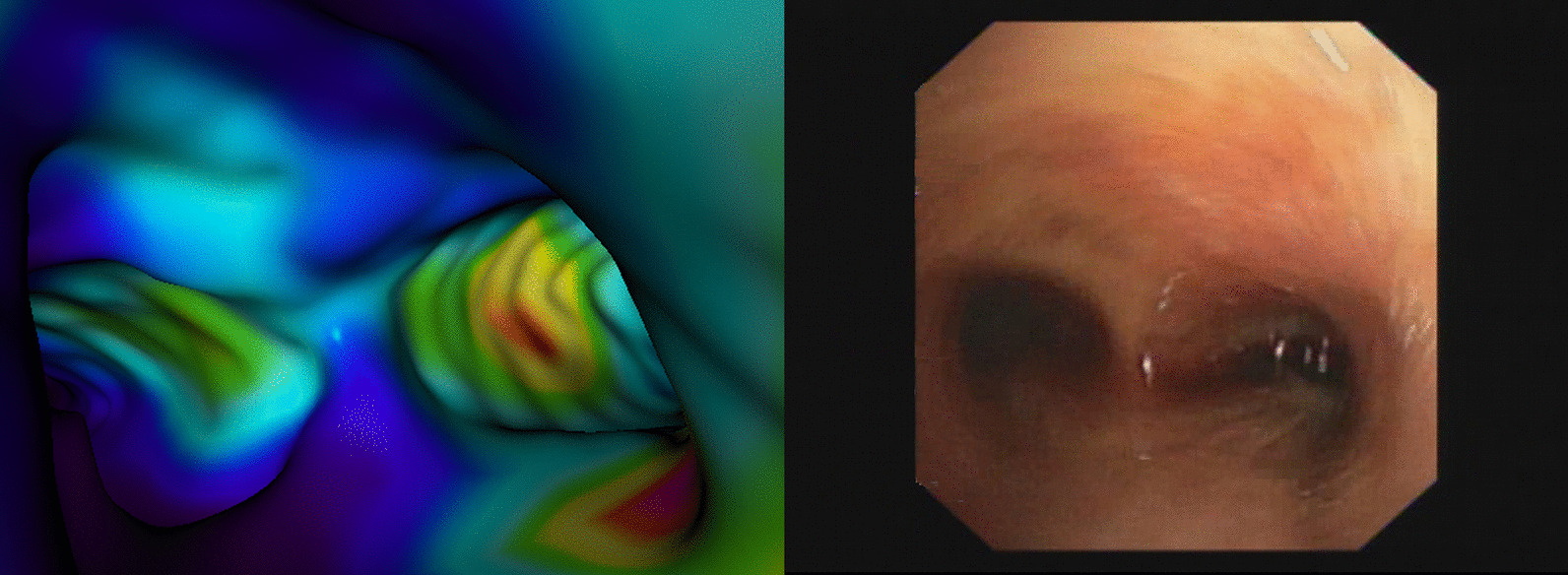
Visualisation of the bifurcation point, as it can be seen from the trachea. The segmented trachea defines the geometry, while the texture originates from the radioactivity accumulated in pixels located 10 mm or closer to the outer surface of the trachea (panel **A**). Real bronchoscopic photograph of the same carinal region (panel **B**)

The bronchial skeleton allows easy traversal of the bronchial tree by a virtual camera to observe the structure and function of the airway or to compare these items with those on the bronchoscope images. Figure 3 displays a snapshot of such a traversal (see a detailed discussion of the figure below). The number of branching points as precisely defined anatomical locations can also be easily counted with the help of this bronchial skeleton. The output of the bronchial tree segmentation served as input for the marching cube algorithm [25] generating a three-dimensional surface model (comprising a high number of triangles) along the sheath of the airway. The number of triangles on this surface model was reduced by a mesh simplification algorithm [26]. Touring along the bronchial tree, the clinician can navigate in space within the luminal structure. Thus, the surface model allows performing VB using the bronchial tree generated by the segmentation algorithm. As mentioned above, the anatomic information was completed with functional data. FDG accumulation reporting on tissue metabolism was projected perpendicularly to the surface elements of the 3D surface model. Accumulation data were summed up from voxels 10 mm from or closer to the surface located outside of the airway. The 10-mm characteristic distance was chosen according to the range of transbronchial needle. Figure 2 shows the 3D surface model of the airway with the superimposed specific functional information derived from the PET image. This metabolic information in Fig. 2 is localised exclusively within the airway contour. Visualisation occurred with an appropriate blending value to make CT voxel intensities negligible.

**Fig. 4:**
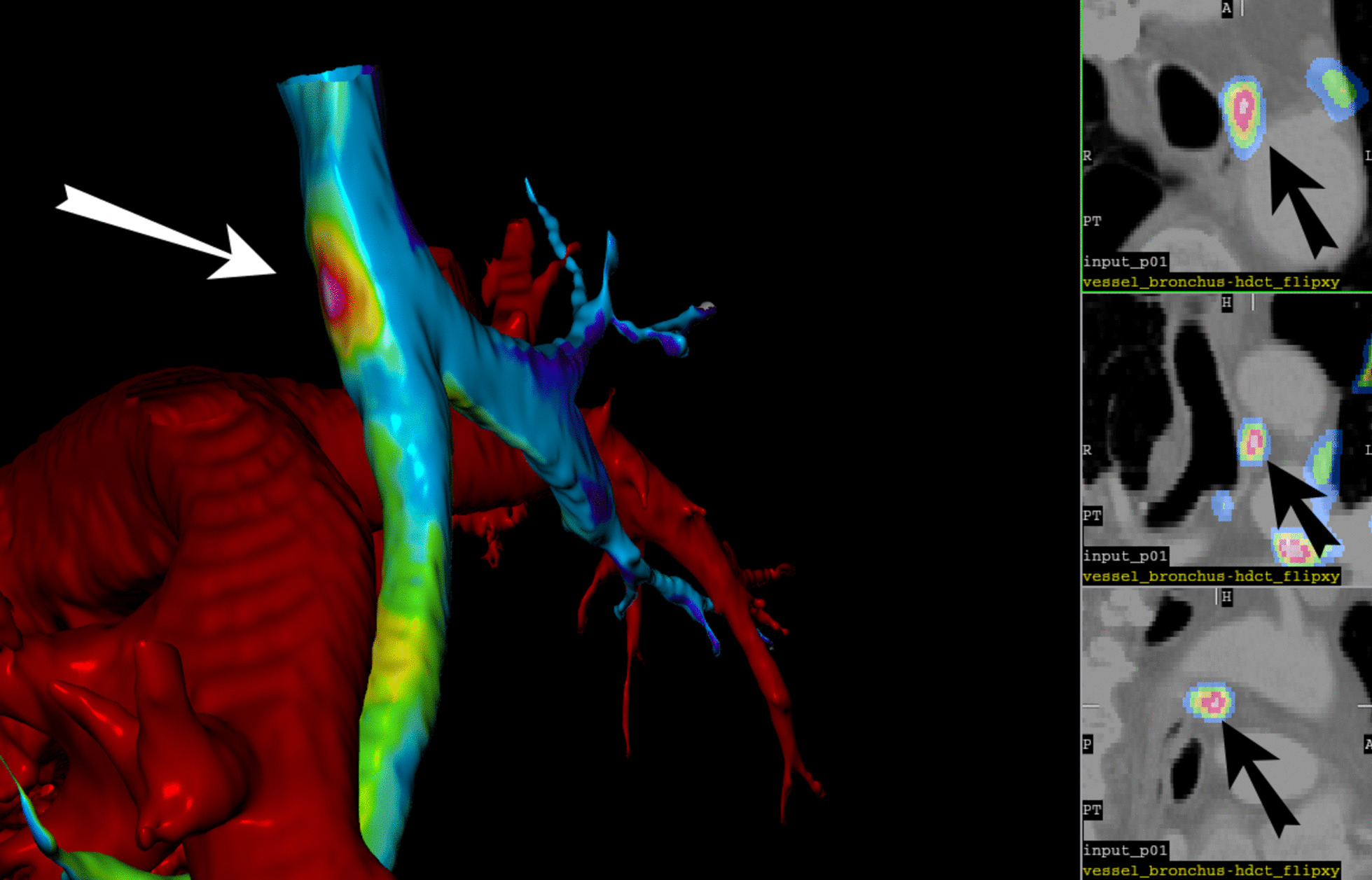
Visualisation of the airway tree and blood vessels from the outside (left) and orthogonal sections in the vicinity of the lesion indicated by the arrow (right). The projected FDG accumulation in the lesion is displayed in spectral scale

Visualisation of the 3D surface model can be performed from both inside (Fig. 3) and outside. Outside visualisation also allows the display of regional blood vessels. To do this, we also segmented the appropriate blood vessels in the same way as described for the airway. Figure 4 shows the final result of visualised structural hdCT segmentations (bronchial tree and blood vessels) and functional data (PET).

#### Registration validation method

**Fig. 5:**
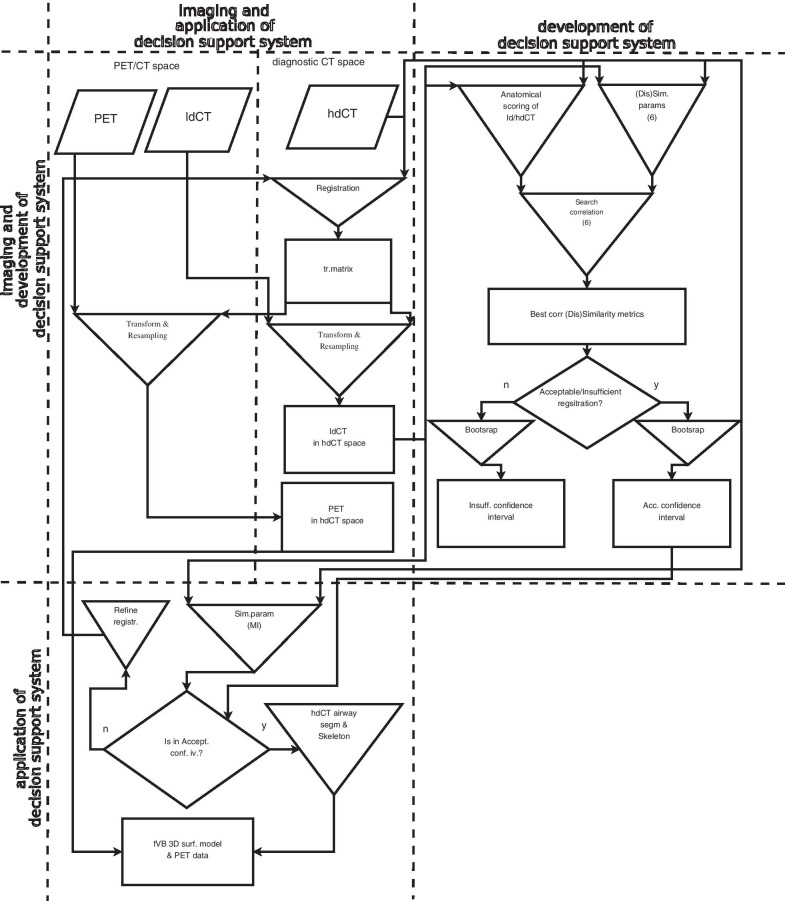
Flowchart of transforming ldCT and PET into diagnostic hdCT space, calculation of intensity based (dis)similarity parameters, distance-based registration’s quality-judging metrics as well as calculation of correlation between intensity- and distance-based parameters. The flowchart also displays the steps necessary to determine the confidence intervals of MI (see detailed explanation in the text)

The high-precision fVB requires high-quality steric and functional information, in other words; both hdCT and PET data are required the latter having been transformed into the hdCT space. The necessary transformation matrix is identical to that applied for the registration of the ldCT image to the hdCT image (Fig. 5). During the realignment processes, we used trilinear interpolation to evaluate registered ldCT and PET images with the same matrix and voxel size as the hdCT was acquired. For this registration, both rigid and non-rigid body transformations were applied due to the eventually different conditions (see “Imaging protocols” section) of the chest CT scannings. Because of the low quality of the ldCT, some registrations of the two CT images were insufficient.

These registrations are unsuitable to proceed towards fVB, while acceptable registrations allow construction of the 3D airway surface model from the hdCT and visualisation of the PET accumulation on the surface model as detailed in “Construction of airway tree and surface model” section. Distinction between acceptable and insufficient registration was made by a radiologist; however, this decision requires careful, meticulous and time-consuming work and is subjective.

We replaced this procedure with an objective method. A numeric parameter, *D* (score) was introduced to characterise the registration quality, derived from 25 distance-type quantities calculated from real distances measured in mm or compared to real measure of anatomical structures (Table 1). A medical imaging specialist physician visually reviewed the hdCT images and identified various parts of the tracheobronchial tree, including the tracheal bifurcation, the origins of the main, lobar and segmental bronchi. The expert marked all the luminal centres in hdCT and checked the localisation of these points on the appropriate registered ldCT image. In case of larger structures (trachea, main bronchi), the distance between the centre of the lumens on the registered hdCT and ldCT image pairs was measured in mm. For each smaller bronchus (columns 5–26 in Table 1), the location of the marked point on the hdCT image was investigated as to whether it was inside the corresponding ldCT bronchial lumen. If so, the distance-type quantity was set to one, while in all other cases to zero.

**Table 1:** Distances, measured in mm, and distance-type quantities of corresponding anatomical localisations (see detailed explanation in the text). Segmental bronchi were labelled according to Boyden’s nomenclature^a^

Pat.	TB [mm]	RMB [mm]	LMB [mm]	RULB	RMLB	RLLB	RB1	RB2	RB3	RB4	RB5	RB6
p01	1.7	0.7	0.9	1	1	1	NA	1	1	0	1	1
p03	0	0	1.1	1	1	1	NA	1	1	1	1	1
p04	0	0.5	0.8	1	1	1	NA	1	1	0	1	1
p05	2	1.1	0	1	1	1	1	1	0	1	0	1
p06	1.9	0	0.4	1	1	1	NA	1	1	1	1	1
p07	1.2	0	0	1	1	1	1	1	1	1	1	1
p08	0	0	0	1	1	1	1	1	1	1	1	1
p09	2.6	0	0	1	1	1	1	1	1	1	1	1
p10	1.9	0	0	1	NULL	NULL	1	0	0	NULL	NULL	NULL
p11	2.4	0	0	1	1	1	1	1	1	1	1	1
p12	1.1	0	0	1	1	1	1	1	1	1	1	1
p13	1.5	0	0	1	1	1	1	1	1	1	1	1
p14	1	0	0	0	0	0	0	0	0	0	0	0
p15	1	1	1	0	0	0	0	0	0	0	0	0
p16	2.5	0	0	1	1	1	1	1	1	1	1	1
p17	0	0	0	1	1	1	1	1	1	1	1	1
p18	0	0	0	NULL	0	0	NULL	NULL	NULL	1	1	1
p20	2.8	0	0	1	1	1	1	1	1	1	1	1
p21	0	0	0	1	1	1	NA	1	1	1	1	NA
p22	0	0	0	1	1	1	1	1	1	1	1	1
p23	0	0	0	1	1	1	1	1	1	1	1	1
p25	2.3	0	0	1	1	1	1	1	1	1	1	1

Pat.	RB8	RB9	RB10	LULB	LLLB	LB1/2	LB3	LB4	LB5	LB6	LB8	LB9	LB10
p01	1	0	0	1	1	0	0	NA	NA	1	1	1	1
p03	1	1	1	1	1	1	1	1	1	1	1	1	1
p04	1	1	1	1	1	1	1	1	1	0	1	1	1
p05	1	1	1	1	1	1	1	1	1	1	1	1	1
p06	1	1	1	1	1	1	0	1	1	1	1	1	1
p07	1	1	1	1	1	1	1	1	1	1	1	1	1
p08	1	1	1	1	1	1	1	NULL	NULL	1	1	1	1
p09	NA	1	1	1	1	1	1	NULL	NULL	1	1	1	1
p10	NULL	NULL	NULL	1	1	1	0	NA	NA	1	1	1	1
p11	0	NA	1	1	1	1	NA	NA	NA	1	1	1	1
p12	1	1	1	1	1	1	1	1	1	1	1	1	1
p13	1	NA	1	1	1	1	1	NA	NA	1	1	1	1
p14	0	0	0	0	0	0	0	0	0	0	0	0	0
p15	0	0	0	0	0	0	0	0	0	0	0	0	0
p16	NA	1	1	1	1	1	1	1	1	1	1	1	1
p17	0	1	1	1	1	1	1	0	0	1	0	1	1
p18	1	1	1	1	1	1	1	1	1	1	1	1	1
p20	1	1	1	1	1	1	1	1	1	1	1	1	1
p21	1	1	1	1	1	1	1	1	1	1	1	1	1
p22	1	1	1	1	1	1	1	1	1	1	1	1	1
p23	1	1	1	1	1	1	1	1	1	1	1	1	1
p25	1	1	1	1	1	NA	NA	NA	NA	1	NA	1	1

^a^*TB* tracheal bifurcation; main bronchi: *RMB* right main, *LMB* left main; right lobar bronchi: *RULB* upper lobe, *RMLB* middle lobe, *RLLB* lower lobe; Right segmental bronchi: *RB1* apical, *RB2* posterior, *RB3* anterior, *RB4* lateral, *RB5* medial, *RB6* superior, *RB8* anterior basal, *RB9* lateral basal, *RB10* posterior basal; Left lobar bronchi: *LULB* upper lobe, *LLLB* lower lobe; left segmental bronchi: *LB1/2* apicoposterior, *LB3* anterior, *LB4* superior lingular, *LB5* inferior lingular, *LB6* superior, *LB8* anterior basal, *LB9* lateral basal, *LB10* posterior basal; *NA* not identifiable in the ldCT (not visually found by an expert); *NULL* cannot be identified in hdCT (because the bronchus is absent (after surgery) or obscured by pathology)

We aimed to identify the (dis)similarity of registered ldCT and hdCT image pairs for both acceptable and insufficient registrations by calculating the numerical value (Fig. 5) of six parameters (Pearson correlation coefficient, mutual information (MI), normalised mutual information, Kullback–Leibler divergence, L1norm, L2norm2 [27]). For the objective characterisation of the quality of the registrations [28], absolute distances were determined between corresponding point pairs of the registered images. We investigated how the (dis)similarity parameters and the measure of the registration's quality of the coherent ldCT and hdCT image pairs correlate*.* Close and low correlations refer to acceptable and insufficient registration, respectively (see the rhomboid-shaped decision symbol in “development of decision support system” part of Fig. 5).

## Results

### Calculation of (dis)similarity parameters

The numerical value of the voxel-based (dis)similarity parameters can be calculated without any spatial limitation using all the voxels (method *α*—see below) or with spatial confinement using only a reduced number of voxels (method *β* and *γ*—see below). For method *γ*, this constraint is more significant.

Parameter values were calculated in three different ways:

- (*α*) masks were not used at all in both ldCT and hdCT images (“no mask”),
- (*β*) the same “functioning lung-mask” was used to both type of the CT images,
- (*γ*) modification of the segmented airway was used as a mask to extend the original airway beyond its boundary up to 10 mm thickness. The modification was completed separately for the ldCT and hdCT images.

### Distance metrics

The parameter values in Table 1 measured in mm were converted (see below) to obtain distance data of the same type. Converted data for the trachea and the two main bronchi (columns 2–4 in Table 1) were set to:

- 1 if the distance between the bronchial centres in hdCT and ldCT was less than 10% of the bronchial diameter (≤ 1.8 mm for trachea, ≤ 1.22 mm for main bronchi).
- 0.5 if this distance was between 10 and 20% of the bronchial diameter ([1.8, 3.6] mm for trachea, [1.22, 2.44] mm for main bronchi).
- 0 if this distance was above 20% of the bronchial diameter (> 3.6 mm for trachea, > 2.44 mm for main bronchi).

The quality *D* of the hdCT–ldCT registration was defined as the weighted sum (score) of the distance-type quantities of the same type. We set the weight factor for the trachea, the main bronchi, the lobular bronchi, the segment bronchi and the sub-segment ones to 16, 7, 3, 1 and 1, respectively (Table 2, first line). Table 2 displays the products of the weight factors and the value of the distance-type quantities recorded in Table 1. Applied weight factors are nearly proportional to the cross section of the appropriate bronchus [29] with scaling based on the idea that a good registration algorithm should unequivocally register large structures acceptably. Thus, *D* provides a score characterising the distance relation of the corresponding anatomical points as a measure of registration’s quality.

**Table 2:** Weighted values of distance-type quantities and their sum (score); we used Boyden’s nomenclature^a^ for abbreviations

Pat.	TB	RMB	LMB	RULB	RMLB	RLLB	RB1	RB2	RB3	RB4	RB5	RB6	RB8
Weight	16	7	7	3	3	3	1	1	1	1	1	1	1
p01	16	7	7	3	3	3	0	1	1	0	1	1	1
p03	16	7	7	3	3	3	0	1	1	1	1	1	1
p04	16	7	7	3	3	3	0	1	1	0	1	1	1
p05	8	7	7	3	3	3	1	1	0	1	0	1	1
p06	8	7	7	3	3	3	0	1	1	1	1	1	1
p07	16	7	7	3	3	3	1	1	1	1	1	1	1
p08	16	7	7	3	3	3	1	1	1	1	1	1	1
p09	8	7	7	3	3	3	1	1	1	1	1	1	0
p10	8	7	7	3	NA	NA	1	0	0	NA	NA	NA	NA
p11	8	7	7	3	3	3	1	1	1	1	1	1	0
p12	16	7	7	3	3	3	1	1	1	1	1	1	1
p13	16	7	7	3	3	3	1	1	1	1	1	1	1
p14	16	7	7	0	0	0	0	0	0	0	0	0	0
p15	16	7	7	0	0	0	0	0	0	0	0	0	0
p16	8	7	7	3	3	3	1	1	1	1	1	1	0
p17	16	7	7	3	3	3	1	1	1	1	1	1	0
p18	16	7	7	NA	0	0	NA	NA	NA	1	1	1	1
p20	8	7	7	3	3	3	1	1	1	1	1	1	1
p21	16	7	7	3	3	3	0	1	1	1	1	0	1
p22	16	7	7	3	3	3	1	1	1	1	1	1	1
p23	16	7	7	3	3	3	1	1	1	1	1	1	1
p25	8	7	7	3	3	3	1	1	1	1	1	1	1

Pat.	RB9	RB10	LULB	LLLB	LB1/2	LB3	LB4	LB5	LB6	LB8	LB9	LB10	Score (*D*)
Weight	1	1	3	3	1	1	1	1	1	1	1	1	62
p01	0	0	3	3	0	0	0	0	1	1	1	1	54
p03	1	1	3	3	1	1	1	1	1	1	1	1	61
p04	1	1	3	3	1	1	1	1	0	1	1	1	59
p05	1	1	3	3	1	1	1	1	1	1	1	1	52
p06	1	1	3	3	1	0	1	1	1	1	1	1	52
p07	1	1	3	3	1	1	1	1	1	1	1	1	62
p08	1	1	3	3	1	1	NA	NA	1	1	1	1	60
p09	1	1	3	3	1	1	NA	NA	1	1	1	1	51
p10	NA	NA	3	3	1	0	0	0	1	1	1	1	37
p11	0	1	3	3	1	0	0	0	1	1	1	1	49
p12	1	1	3	3	1	1	1	1	1	1	1	1	62
p13	0	1	3	3	1	1	0	0	1	1	1	1	59
p14	0	0	0	0	0	0	0	0	0	0	0	0	30
p15	0	0	0	0	0	0	0	0	0	0	0	0	30
p16	1	1	3	3	1	1	1	1	1	1	1	1	53
p17	1	1	3	3	1	1	0	0	1	0	1	1	58
p18	1	1	3	3	1	1	1	1	1	1	1	1	50
p20	1	1	3	3	1	1	1	1	1	1	1	1	54
p21	1	1	3	3	1	1	1	1	1	1	1	1	60
p22	1	1	3	3	1	1	1	1	1	1	1	1	62
p23	1	1	3	3	1	1	1	1	1	1	1	1	62
p25	1	1	3	3	0	0	0	0	1	0	1	1	49

^a^*TB* tracheal bifurcation; Main bronchi: *RMB* right main, *LMB* left main; right lobar bronchi: *RULB* upper lobe, *RMLB* middle lobe, *RLLB* lower lobe; right segmental bronchi: *RB1* apical, *RB2* posterior, *RB3* anterior, *RB4* lateral, *RB5* medial, *RB6* superior, *RB8* anterior basal, *RB9* lateral basal, *RB10* posterior basal; Left lobar bronchi: *LULB* upper lobe, *LLLB* lower lobe; Left segmental bronchi: *LB1/2* apicoposterior, *LB3* anterior, *LB4* superior lingular, *LB5* inferior lingular, *LB6* superior, *LB8* anterior basal, *LB9* lateral basal, *LB10* posterior basal. The last column (score-*D*) is the weighted sum of the displayed distance-type data

### Correlation calculation

Linear model-based regression calculations (MASS R-package [30]) were performed to determine whether there is any (dis)similarity parameter, correlating with the value of *D*. The *ϱ* correlation coefficients are listed in Table 3. Numerical values of *D* and (dis)similarity parameters are displayed in Table 4. (Dis)similarity parameters giving significant correlation (*p* < 0.05) with the score values are shown in Table 5 in case of *γ* masking technique.

**Table 3:** Correlation coefficients (*ϱ*) between the distance metrics and the intensity-based (dis)similarity parameters (calculated in three different manners: *α*, *β*, *γ*; see details in the text)

Masking	(dis)similarity parameter					
	Pearson (s)	MI (s)	NMI (s)	HKL (d)	L1norm (d)	L2norm2 (d)
*α*	0.15	0.02	0.02	− 0.07	0.25	0.22
*β*	0.38	0.56	0.5	0.45	− 0.04	− 0.09
*γ*	0.4	**0.6**	0.51	− 0.02	0.07	0.09

*MI* mutual information, *NMI* normalised mutual information, *HKL* Kullback–Leibler divergence, *L1Norm* L_1_ norm or Manhattan norm, *L2norm2* square *L*_2_ norm or square Euclidean distance, *(s)* similarity parameter, *(d)* dissimilarity parameter (data are rounded to two significant digits, the highest correlation coefficient is emphasized in bold text)

**Table 4:** Calculated values of the quality of registration (score) and the (dis)similarity parameters of corresponding ldCT and hdCT image pairs in case of *γ* masking technique (data are rounded to two significant digits)

Pat	Score	(dis)similarity parameter					
		Pearson	MI	NMI	HKL	L1norm * 1e−7	L2norm2 * 1e−10
p01	54	0.86	0.44	1.21	0.44	20.55	13.61
p03	61	0.25	0.25	1.1	0.74	4.1	2.07
p04	59	0.74	0.44	1.21	0.62	15.1	10.4
p05	52	0.32	0.32	1.11	0.55	8.18	5.42
p06	52	0.31	0.31	1.11	0.74	5.89	3.45
p07	62	0.24	0.25	1.09	0.78	4.36	2.34
p08	60	0.28	0.28	1.1	0.8	4.71	2.45
p09	51	0.19	0.19	1.08	0.85	3.11	1.41
p10	37	0.19	0.2	1.09	0.76	4.75	2.5
p11	49	0.24	0.22	1.1	0.79	6.8	4.1
p12	62	0.47	0.47	1.17	0.79	5.35	2.88
p13	59	0.17	0.18	1.07	0.68	6.47	4.21
p14	30	0.15	0.15	1.06	0.75	7.03	4.15
p15	30	0.1	0.1	1.04	0.75	4.67	2.34
p16	53	0.41	0.31	1.13	0.69	10.52	6.87
p17	58	0.34	0.34	1.12	0.69	7.12	4.62
p18	50	0.2	0.2	1.08	0.84	3.83	1.58
p20	54	0.21	0.21	1.08	0.7	8.24	5.2
p21	60	0.43	0.43	1.17	0.81	2.47	1.08
p22	62	0.41	0.41	1.19	0.8	3.97	1.75
p23	62	0.22	0.22	1.08	0.62	10.57	7.32
p25	49	0.26	0.26	1.09	0.44	7.8	5.08

**Table 5:** Values of the *p*, estimated correlation of score and similarity parameters, and the lower and upper limits of confidence intervals (with *γ* masking technique)

(dis)similarity parameter	*p*	Corr	CI_lower	CI_upper
Pearson	0.0673	0.3971	− 0.0295	0.7013
MI	0.0033	0.5981	0.2360	0.8143
NMI	0.0150	0.5115	0.1146	0.7676

Data are displayed for similarity parameters giving significant correlation with the score values (*p* < 0.05)

The analysis revealed that the MI displayed the closest correlation with the anatomy-based distance metrics (*ϱ* = 0.5981, *p* = 0.0033).

### Confidence intervals

**Fig. 6:**
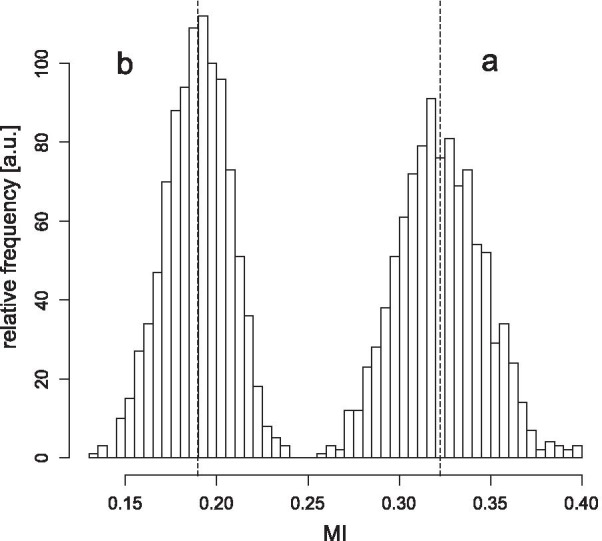
MI distribution calculated from the bootstrapped samples (a: for the accepted registrations; b: for the rejected registrations). A vertical dashed line indicates the mean of MI values for both parts of the distribution (0.19 and 0.324)

We aimed to establish whether the numerical value of the MI can suggest acceptable vs insufficient ldCT–hdCT lung image pair registration classification is applicable to any patient. To answer this question, one should draw a large number of samples from the population of interest. However, the time required to gather this vast amount of data is extensive, and the procedure is labour intensive and too expensive. Instead, we used the frequently applied bootstrap statistical method [31, 32] providing a bootstrapped distribution of the MI statistics, which approximates the MI distribution in the whole population. For this purpose, we divided our 22 cases into two parts according to the quality of the registration as measured by score value (the higher the better) and constructed the bootstrapped MI distribution separately for the acceptable (15 subjects) and insufficient (7 subjects) subsets assigned to the 22-member scanned population (Fig. 6).

The numerical value of MI is image pair dependent, and its value fluctuates from sample to sample. The fluctuation can be characterised by confidence intervals. If one trims off a small percentage (e.g. 2.5%) from both the lower and upper end of the distribution, the remaining interval includes 95% of all the cases.

The distribution displayed in Fig. 6 indicates that the registrations can be separated into two groups based on MI values. We also examined the confidence intervals of the bootstrapped MI for insufficient and acceptable registrations. The 95% confidence intervals were [0.15, 0.22], [0.28, 0.37], respectively.

For the subpopulation acceptable and insufficient registration, we separately examined how the mean of their bootstrapped distributions (Fig. 6) differed from the mean of the appropriate scanned populations (bias). Values obtained:

$$^{{{\text{MI}}}} {\text{bias}}_{{{\text{acc}}}} = 0.00018,\;^{{{\text{MI}}}} {\text{bias}}_{{{\text{insuf}}}} = - 0.00080.$$

Consequently, in case of any new registration, a calculated MI value within the [0.28, 0.37] (mean: 0.324) or the [0.15, 0.22] (mean: 0.19) interval would suggest acceptance or rejection, respectively, with 95% confidence serving as an aid for the radiologist. In case of insufficient registration, the registration has to be repeated with slightly different conditions. MI values between the two confidence intervals (values between 0.22 and 0.28) also indicate the necessity of a new registration.

## Discussion

VB is frequently used as a potent diagnostic modality. Application of fVB is even more advantageous because along with the morphological details, it also visualises the areas of malignancy and tumour growth and the tumorous tissue both in- and outside the bronchus. The functional information enables early diagnosis as the metabolic change within the infiltrated region precedes the development of anatomical changes. Complementing anatomical information with functional data would assist the tissue sampling from the right location. The glucose accumulation map delivered by PET scan can also help in the assessment of the tumour aggressiveness, therapeutic response and recurrences. The incorporation of functional information into the practice of virtual diagnostics is generally done by registering the PET image to the bronchoscope video image. Thus, the physician does not have to solve the “merging” of a pre-viewed, digitally generated hdCT–PET image and a video image viewed during tissue sampling. Fortunately, there are working algorithms [33, 34] that display the two 3D images (hdCT–PET and digitised bronchoscope video image) in a single merged image.

The quality of the fVB is improved if we use the higher-resolution hdCT instead of ldCT images (Fig. 1). The use of hdCT is justified because it can map structures not resolved by ldCT. For high-quality fVB, the registration of the ldCT and hdCT images is indispensable, because the transformation matrix of this registration is necessary to fit the PET and hdCT images anatomically correctly.

In the initial phase of our project, a radiologist decided whether ldCT–hdCT registration was acceptable or not. However, the judgement of a radiologist expert is subjective and difficult to scale. Therefore, we were looking for a solution characterising registration quality objectively and independently of the radiologist. Numeric value of (dis)similarity parameters of registered image pairs can be calculated easily, so it is worth looking at whether there are any of them correlating with the measure of the registration quality (scoring of the appropriate structures’ fit by an expert). In case of a close correlation, a simple software procedure can substitute the radiologist’s categorisation of any new ldCT–hdCT registration. Rohlfing has shown that distance-type errors should be used for reporting reliable registration errors [28]. As *D* provides a distance-type characterisation of the point pair's set of the registered images, a parameter correlated with *D* maintains approximately the same characterisation. Application of this software procedure is advantageous due to its simplicity compared to the labour-intensive calculation of *D*. At the same time, it might give a somewhat looser characterisation of registration quality.

For software judgement of the registration’s quality, we used parameters to show the similarity (the larger, the better) and the dissimilarity (the less, the better) in the voxel intensity data of the fitted images. For this purpose, the easily calculable Pearson correlation coefficient, mutual information, normalised mutual information, Kullback–Leibler divergence, L1norm and L2norm2 were chosen. Out of these parameters, those providing a close correlation with the distance-based *D* registration quality can be used to classify the registration as acceptable or insufficient. Contrary to expectations, we found a close correlation in only one case, suggesting that the (dis)similarity parameters define the concept of similarity and dissimilarity in different ways.

The categorisation can be performed using confidence intervals of the (dis)similarity parameters. Registrations with MI parameter value falling within the confidence interval “*a*” and “*b*” of the distributions (Fig. 6) are considered acceptable and insufficient, respectively. Parameter values falling within the “*b*” confidence interval indicate that the registrations are to be repeated under more tightly controlled conditions.

Substitution of ldCT images with diagnostic hdCT images will further strengthen the successful applicability of the fVB and improve its clinical achievements. A disadvantageous feature of the developed procedure is that it has to be reconstructed (the confidence intervals have to be recalculated) in case of any change in the imaging equipment, segmentation or registration protocols.

## Conclusion

Patients undergoing bronchoscopy have previously undergone a series of examinations, including in the majority of cases CT and PET examinations. The latter investigation is generally performed in the form of PET/ldCT. Many PET centres usually do not even perform hdCT testing because the indication is usually tumour search. If the whole investigation process indicates that bronchoscopy is required, a prospective hdCT test either alone or with a concomitant PET scan would involve an extra dose of radiation, which would increase the likelihood of developing a second tumour.

An automatic pipeline was worked out for virtual bronchoscopic examinations based on both PET and hdCT data using retrospective scans to avoid additional prospective HRCT study with the extra radiation dose involved. Accurate manual verification of the quality of ldCT–hdCT registration, which is a critical element of the procedure, would traditionally require time-consuming radiologist expert work, for the replacement of which we have developed an automated procedure.

The proposed procedure will likely increase the diagnostic yield of fVB as the added value of HRCT and PET methods is difficult to question. The precise visualisation of functional information can decrease the number of biopsies taken from inadequate locations. Displaying the blood vessel system (Fig. 4) can reduce the risk of artery or vein perforation [35].

Results of fVB examinations supported by PET and hdCT imaging have not been published so far to our best knowledge; thus, numerical data on how our method improves the current workflow can only be obtained by performing a large number of studies.

## Data Availability

The datasets analysed during the current study are not publicly available due to containing information that could compromise research participant privacy but are available from the corresponding author on reasonable request.

## Appendix

Our M-SEGM segmentation algorithm is semi-automatic (Multi-SEGMentation: M-SEGM). The user has to specify the placement of the starting point (seed point) of the segmentation within the desired region (e.g. inside the trachea) and the intensity range with low and high limits as the homogeneity criterion. This algorithm attaches voxels one by one to the previous voxel set performing iterations, starting from the seed point. The intensity of the last attached voxel is in the range specified by the homogeneity criterion *(airway: [− 1000, − 950], parenchyma: [− 800, − 400], contrast-enhanced blood vessel**: **[200,400]**)*. Voxel addition continues until the last voxel is not yet tested, followed by the stop of the process and the generation of the segmented mask.

## Abbreviations

CT: Computed tomography
PET: Positron emission tomography
FDG: Fluorodeoxyglucose
VB: Virtual bronchoscopy
hdCT: High-dose CT
ldCT: Low-dose CT
fVB: Functional virtual bronchoscopy
HRCT: High-resolution CT
kVp: Kilovolt peak
AEC: Automatic exposure control
M-SEGM: Multi-SEGMentation
M3I: Multi-modal medical imaging
Pearson: Pearson correlation coefficient
MI: Mutual information
NMI: Normalised mutual information
HKL: Kullback–Leibler divergence
L1norm: Manhattan distance
L2norm2: Euclidean distance
ANOVA: Analysis of variance
